# The Impact of Online Learning on Physical and Mental Health in University Students during the COVID-19 Pandemic

**DOI:** 10.3390/ijerph19052966

**Published:** 2022-03-03

**Authors:** Yu-Hsiu Chu, Yao-Chuen Li

**Affiliations:** Department of Physical Therapy, China Medical University, No. 100, Section 1, Jingmao Road, Beitun District, Taichung City 406040, Taiwan; yhchu@mail.cmu.edu.tw

**Keywords:** COVID-19, learning, physical activity, psychological distress, student life stress, emerging adults

## Abstract

Higher education organizations have been influenced by the COVID-19 pandemic. During school closures, online teaching and learning has become a new routine that may lead to changes in lifestyles and adversely affect university students’ health. Therefore, this study was to understand the potential impact of online learning on physical and mental health by investigating the differences in physical activity, psychological distress, and student life stress in Taiwanese university students between the in-class learning and online learning periods. A total of 181 students were recruited from a local university. All participants were requested to complete an online survey and self-report physical activity, psychological distress, and life stress in the in-class learning and online learning periods, respectively. The results indicated a significant reduction in physical activity of various intensities (*p* < 0.05). Specifically, male university students showed a greater decrease in vigorous physical activity compared to their female peers. Yet, there was no significant increase in psychological distress and life stress from the in-class learning period to the online learning period. In summary, physical activity drastically reduces during the online learning period in Taiwanese university students. Notably, male students may be at greater risk of insufficient participation in vigorous physical activity.

## 1. Introduction

The coronavirus disease 2019 (COVID-19) has become one of the most serious health issues worldwide since the first case was confirmed in 2019 and the global outbreak occurred in 2020 [1]. According to the World Health Organization (WHO), as of 1 September 2021, there have been more than 218 million confirmed cases, with more than 4 million deaths reported by more than 200 countries [2]. In order to effectively control the spread of COVID-19, many policies have been adopted, such as social distancing, border control, and school closures [2,3]. In spite of the fact that vaccinations have been introduced to tackle the COVID-19 pandemic [2], the pandemic has undoubtedly, significantly changed our lives and behaviors in a variety of aspects, such as participation in physical activity, social interactions with friends, and teaching and learning at education organizations [4,5,6,7]. Consequently, our physical and mental health might be influenced and deteriorated as a result of these unusual changes.

Prior research has identified the adverse effect of COVID-19 on emerging adults’ physical and mental health due to social distancing and other measures [6,8]. A large-scale cross-sectional study that analyzed data collected from 18 countries investigated the global changes in exercise behaviors and mood and found that, during the COVID-19 pandemic between March and May 2020, 23.7% of respondents reported a decrease in exercise frequency, and there was a nearly linear relationship between exercise frequency and mood during the pandemic, indicating that individuals who exercised more frequently may have had a better mood [4]. Furthermore, a review on the epidemiology of mental illness synthesized the existing evidence and identified greater risks for depression, anxiety, or post-traumatic stress disorder in the general population, as well as in patients with COVID-19 and health providers [9], whereas a systematic review of 12 articles found a higher prevalence of depression or anxiety symptoms in children and adolescents aged between 8 and 18 years [8]. Therefore, physical and psychological health in various populations might be inevitably affected during a lockdown or school closure [10].

Lockdown and/or school closure may bring significant impacts on higher education, as novel teaching and learning approaches, such as digital online learning or virtual classroom, were introduced during these unprecedented situations [3,5]. Specifically, the adaptation to these novel approaches may further increase university students’ anxiety and decrease their motivation and confidence toward personalized learning due to technological barriers during the online learning period [11]. In addition, as university students have been found to be at greater risks for insufficient participation in physical activity [12], a recent systematic review also found a further reduction in physical activity levels at any intensity in this specific population during lockdown [13].

It is evident that there is an intertwined relationship between physical activity and mental health [14]. Previous findings have shown that prolonged sitting periods or sedentary lifestyles are strongly associated with increased risks for non-communicable diseases, such as obesity or diabetes, in adults [15,16]. These diseases may commonly coexist with or lead to mental health problems [17,18]. This is also the case during the COVID-19 pandemic, as a previous study identified a positive association between higher exercise frequency and better mood in Taiwanese adults [19]. As a result of school closures, university students may be more susceptible to physical inactivity and mental illness during the online learning period [10,11,13]. Therefore, it is of particular urgency and importance to draw attention to both physical and mental health in this population.

When the first COVID-19 case was confirmed on 21 January 2020, there was an outbreak in Taiwan between January and April with 55 domestic cases [20]. By taking strict and effective strategies [21], the outbreak was soon subsided, and there was no confirmed domestic case for seven consecutive months from May to November of 2020 [20]. Most universities remained open during the first COVID-19 outbreak, even though some courses were simultaneously delivered in the classroom and online; that is, the students could either go to the classroom or stay home to learn online. However, when the second COVID-19 outbreak occurred in May 2021, the Level 3 epidemic alert for the COVID-19 was announced by the Taiwan government to reduce community transmission [22]. Meanwhile, the Ministry of Education took a much stricter strategy to prevent the pandemic: all public and private education organizations were requested to be shut down. Therefore, since 19 May, all schools and educational institutions have suspended in-person instruction, and courses have been shifted to online [23].

Although school closures are a must-do measure in response to the COVID-19 pandemic, this may impose detrimental effects on individuals’ physical and mental health along with other regulations, such as social distancing, no public gathering, and no dine-in at restaurants [21,22,23]. However, limited research has been conducted to evaluate the impact of the change in the teaching and learning approach on physical and mental health in university students, and evidence is scarce regarding the differences in physical and mental health between the in-class and online learning periods. To this end, this study was conducted to better understand the impact of school closures during the COVID-19 pandemic and investigate the differences in physical activity, psychological distress, and student life stress in university students between the in-class learning period and the online learning period. Specifically, it was hypothesized that, when compared with the in-class learning period, university students would be less likely to engage in physical activity and experience more life stress and psychological distress during the online learning period.

## 2. Materials and Methods

### 2.1. Participants and Procedures

This was a cross-sectional study approved by the China Medical University and Hospital Research Ethics Committee (CMUH 110-REC3-108). Undergraduate and graduate students at a medical university in central Taiwan were recruited through postings on social media in July 2021. Any potentially eligible participants who saw the recruitment advertisement could access the link of the anonymous web-based survey, which included a consent form, demographic information, and questionnaires concerning physical activity, psychological distress, and student life stress. Nevertheless, they were requested to confirm their eligibility before answering any questions, including: (1) they are currently students at the university, and (2) they are able to read the questions in Mandarin. In addition, students in the final year of their program were excluded from this study, as most of them had to complete an internship at off-campus medical institutes. A total of 742 students accessed and anonymously completed the online survey. However, there were 48 students who were ineligible and 413 students who did not complete all questions. Finally, there were only 181 students (24.4%) who completed all the questions (124 females and 57 males; mean age = 21.82 ± 3.14 years). It took approximately 15 to 20 min to complete the survey. Notably, as this was a cross-sectional study that was conducted after the second outbreak of the COVID-19, all participants completed the online survey only once with careful instructions regarding their answers to health behaviors or status during the in-class learning and online learning periods.

### 2.2. Measures

#### 2.2.1. Physical Activity

The Taiwanese version of the International Physical Activity Questionnaire—Short Form (IPAQ-SF, available at https://sites.google.com/site/theipaq/ (accessed on 1 July 2021)) was used in this study to collect data on physical activity at various intensities [24,25]. The IPAQ-SF has been validated to show acceptable repeatability (75% of Spearman’s ρ > 0.65), reasonable agreement with the Long Form, and fair to moderate criterion validity against accelerometers [25], while the Taiwanese version also showed good test-retest reliability (Spearman’s ρ = 0.67) and good agreement with the Taiwan version of the Long Form (Spearman’s ρ = 0.86) [26]. Participants were requested to recall and report their time spent in light physical activity (LPA; i.e., walk), moderate physical activity (MPA), and vigorous physical activity (VPA) in the past 7 days before the day when online learning had begun (i.e., in-class learning period) and in the past 7 days (i.e., online learning period), respectively. The times of total physical activity (TPA) and moderate-to-vigorous physical activity (MVPA) were also calculated and recorded for each period. Compliance with WHO recommendations for adults (i.e., at least 150 min per week) was then calculated based on their MVPA [27].

#### 2.2.2. Psychological Distress

The Mandarin version of the Kessler-6 (K6) scale (available at https://www.hcp.med.harvard.edu/ncs/k6_scales.php (accessed on 1 July 2021)) was used to measure psychological distress of undergraduate and graduate students in this study [28]. The K6 scale has been validated to show excellent internal consistency (Cronbach’s α = 0.89 to 0.92) and discriminative validity [28], and the Mandarin version has also been validated in an international survey study [29]. It includes six items measuring different emotional status (e.g., nervous, hopeless, and depressed) during the past 30 days. Participants were asked to answer each question using a 5-point Likert scale, ranging from 0 (none of the time) to 4 (all of the time) and yielding a total score of 0 to 24, with a higher score indicating a higher level of psychological distress. A score of 12 was usually used as the cut-off for serious mental illness [28]. Similarly, they needed to recall their psychological status and complete the questionnaire for the in-class and online learning periods, respectively.

#### 2.2.3. Student Life Stress

The Student Stress Inventory (SSI) was developed to assess stress in university students and was used in this study with permission obtained from the authors. The SSI has been validated to show moderate to good content validity, which was approved by nine experts, and good internal consistency (Cronbach’s α = 0.62 to 0.86) [30]. In spite of the insufficient sample size, our preliminary analysis showed excellent internal consistency in this study (Cronbach’s α = 0.93 for both the in-class and online learning periods). The SSI consisted of 40 questions that were divided into four subscales with 10 items in each subscale, including physical stress, interpersonal relationship, academic stress, and environmental stress. Participants were asked to read each question and report how often they experienced the symptom, situation, or problem using a four-point Likert scale (1 = never, 2 = somewhat frequent, 3 = frequent, and 4 = always). The original version did not require the respondents to answer the questions based on their experience within a specific time frame. However, in order to better help our participants recall their experience, this study asked them to consider their situation in the past 7 days before online learning had begun and in the past 7 days when they responded to the questionnaire. Based on the manual, the total score, ranging between 40 and 160, was calculated by summing the item scores, whereas the subscale scores were calculated by averaging the item scores in each subscale. A higher score indicated a higher level of life stress.

### 2.3. Data Analysis

Data were first visually inspected to identify any potential outliers. While large variations were specifically found for variables with respect to physical activity, those outliers (∣z∣ score ≥ 3) were further excluded from data analysis [31]. SPSS 22.0 for Windows (Armonk, NY, USA: IBM Corp) was used to conduct the statistical analyses. Descriptive statistics were expressed as mean ± standard deviation (SD) or a percentage to describe the demographic information of our participants. Furthermore, a repeated-measures ANCOVA with sex as the fixed factor was conducted to assess the difference in psychological distress between the in-class and online learning periods, whereas two repeated-measures MANCOVAs with sex as the fixed factor were conducted to assess the differences in physical activity and student life stress, respectively. Taking into account the potential confounding effect [9], the school of study, the year of study, and the medical diagnosis regarding physical or mental health were entered as covariates in all analyses. Statistical significance was set at α < 0.05.

## 3. Results

### 3.1. Sample Characteristics

A total of 181 university students participated in this study. As shown in Table 1, the majority of them were second-year undergraduate students (*n* = 58, 32.04%) and studied at the School of Health Promotion (e.g., nursing or physical therapy; *n* = 60, 33.15%). More than 50% of the participants had a normal weight status (*n* = 119). In addition, there were 20 participants (11.05%) who reported a medical diagnosis of physical or psychiatric problems that may impact their physical (e.g., physical fitness or physical activity) or mental health (e.g., anxiety or depression).

During the in-class period (Table 2), there were 86 participants (55 females, 63.95%) who reported meeting physical activity recommendations, while compliance decreased to 24.31% (*n* = 44; 35 females, 79.55%) during the online learning period. In addition, 56 participants (39 females, 69.64%) were identified as having a serious mental illness based on the results of the K6 during the in-class learning period; however, there were 49 participants with a serious mental illness (33 females, 67.35%) during the online learning period.

### 3.2. Physical Activity

**Figure 1:**
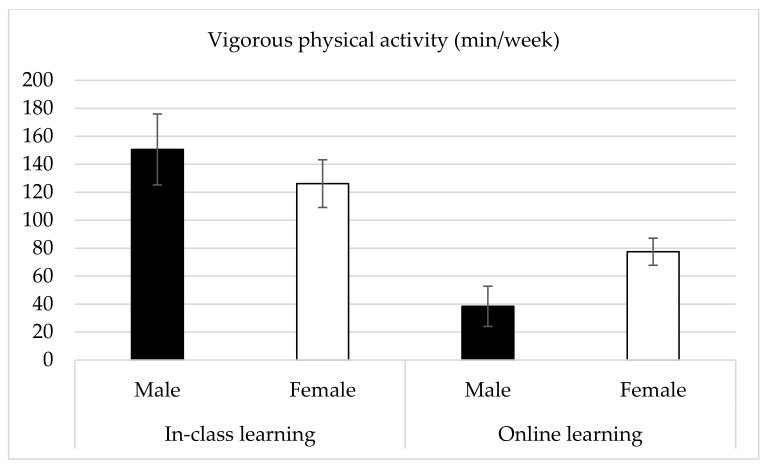
Vigorous physical activity in males and females during in-class and online learning periods.

The results show a statistically significant period effect on physical activity at different intensities (*p* < 0.05) and total physical activity (*p* < 0.001, Table 3), indicating that physical activity levels were lower during the online learning period. No sex effect was found on any physical activity variables; however, a significant interaction of period by sex was found on VPA (*F*_(1, 157)_ = 4.83, *p* < 0.05, $\eta_{p}^{2}$ = 0.03). The result is graphed in Figure 1 that VPA was found to decrease from the in-class learning period to the online learning period in both male and female students, but the extent to which VPA declined was larger in males than in females.

### 3.3. Psychological Distress

The result did not show statistically significant period (*F*_(1, 176)_ = 0.01, *p* > 0.05, $\eta_{p}^{2}$ < 0.01) and sex effects (*F*_(1, 176)_ = 0.28, *p* > 0.05, $\eta_{p}^{2}$ < 0.01) on psychological distress. In addition, there was no significant interaction of period by sex on psychological distress (*F*_(1, 176)_ = 1.06, *p* > 0.05, $\eta_{p}^{2}$ = 0.01).

### 3.4. Student Life Stress

The same as to the result of psychological distress (Table 4): neither period effect nor sex effect was found on the subdomains of student life stress and overall life stress (*p* > 0.05). In addition, there was no significant period by sex interaction on student life stress (*p* > 0.05).

## 4. Discussion

The present study investigated the potential impact of school closures on physical and mental health in university students by comparing physical activity levels, psychological distress, and student life stress between the in-class learning period and online learning period during the COVID-19 pandemic. To the best of our knowledge, this study may be the first to investigate this issue in Taiwanese university students. Our hypotheses were partly supported, showing that university students participated in physical activity significantly less in the online learning period compared with the in-class learning period. Nevertheless, their mental health was not influenced.

As the COVID-19 pandemic may significantly change our health behaviors [32], this study specifically identified that university students’ physical activity levels were more affected during the online learning period. While previous studies have shown that 64.6–79.6% of university students could maintain a level of health-enhanced physical activity during lockdown [33,34], there were only 24% of Taiwanese students in this study who could accumulate at least 150 min of MVPA per week during the online learning period. Furthermore, consistent with a recent systematic review showing a reduction in physical activity in university students during lockdown [13], our participants were significantly physically inactive and only engaged in physical activity for less than 50% of the time that they previously spent during the in-class learning period. As all classroom-based courses had been moved online and accessibility to exercise facilities at universities had become impossible during school closures, this may have hindered university students from maintaining their physical activity routines and regularly participating in exercise or sports [3,35]. Instead, their time spent in physical activity may have been replaced by sedentary behaviors, as online learning may request a large amount of time using electronic devices [36,37]. Furthermore, other measures taken during the online learning period (i.e., the Level 3 Control Stage) could simultaneously and significantly reduce their opportunities to engage in daily physical activities, including closure of all places of entertainment and leisure, social distancing, and domestic travel restrictions [38,39].

While prior research has consistently found a decline in total physical activity during the COVID-19 pandemic or school closures, little attention has been drawn to its impact on different intensities of physical activity [10,36,37,40]. In line with prior research [36,37,40,41], our finding showed that the decline in physical activity was more remarkable in those activities with lower intensity (i.e., walking in this study). Evidence consistently indicates that a decline in physical activity during a lockdown or school closure would enlarge with a decrease in the intensity. This finding may not be surprising, as university students may commute to and from the university by walking or public transportation. Due to the cancellation of in-class courses, this may deprive their walking time [37,40], thereby leading to a drastic decrease in LPA.

It is also worth noting that sex may play an important role in the change in physical activity from the in-class learning period to the online learning period during the COVID-19 pandemic [41]. Rodriguez-Larrad and colleagues found that there was a greater reduction in higher intensity physical activity (i.e., MVPA) in male university students during the confinement in 2020. This is also the case in our study. Specifically, a greater decrease was found in VPA in males (74%) compared to their female peers (39%). Furthermore, while males spent more time on VPA during the in-class learning period (male: 150.62 min/week; female: 126.17 min/week), their VPA time became even lower than females during the online learning period (male: 38.46 min/week; female: 77.48 min/week). This finding implies that the types of VPA in which male students choose to participate may be more directly affected by school closures, such as exercising at university gyms or playing intramural sports [41]. Therefore, when these activities or facilities may be inaccessible or unavailable during the online learning period, male students are less likely to engage in these vigorous physical activities. On the other hand, female students may participate in different types of VPA that are less affected by school closures, such as computer applications or exergames [41]. However, as this study did not investigate the types of physical activity in both periods, further research is warranted to better understand the underlying mechanisms.

On the contrary, while the majority of research has identified higher levels of psychological distress and/or life stress in university students during a lockdown or school closure in a similar cultural context [42,43], this study did not find a difference in psychological distress and student life stress between the in-class learning and online learning periods. We argue that this discrepancy in findings may be due to the timing when the study had been undertaken. The existing evidence showing mental illness in university students during a lockdown or school closure was obtained in studies that were completed in 2020 when the COVID-19 pandemic first occurred and overwhelmingly spread around the world. As this was a new disease that has not been observed in human history, individuals may have felt anxious and depressed toward the huge changes in their lives caused by this uncertain situation and, consequently, suffered from mental health problems. Nevertheless, when this study was conducted during the second outbreak in Taiwan in 2021, students may have been mentally ready for any change in teaching and learning approaches, as they had experienced such changes during the first outbreak in 2020. Therefore, no deterioration in mental health was found in this study. However, as physical inactivity could exert an adverse effect on mental health [44,45,46], this calls for the need for longitudinal research that could track the long-term impact of insufficient participation in physical activity on mental illness in this population.

One of the limitations of this study is the small sample size due to the low completion rate of the online survey. As this study was conducted during the online learning period, face-to-face recruitment was not possible. This, in turn, limited our ability to ensure the quality of participants’ responses. For instance, some students completed part of the survey or only missed a few items of the questionnaire. This led us to exclude some data for further analysis. Another limitation is the representation of our sample. As a majority of the participants were recruited from the School of Health Promotion, there may be difficulty in generalizing the results. A more balanced, stratified sampling is needed for further research. Third, even though we initiated this study soon after school closures (May 2021), we waited for a longer time to receive approval from the Research Ethics Board. This delay may have caused measurement errors due to recall bias when the participants were asked to report their physical activity and the status of mental health. It is strongly recommended that educational institutions should regularly record students’ physical and mental health during this unprecedented period. By doing so, we could more accurately evaluate the long-term impact of the COVID-19 on physical and mental health.

## 5. Conclusions

To sum up, when physical activity significantly reduces during the online learning period in both male and female university students, their mental health is not affected by school closures. Furthermore, much attention should be drawn toward the male population, as their participation in higher intensity physical activity (i.e., VPA) is specifically impacted compared to females. These insights provide health professionals and universities with valuable information for developing preventive strategies targeting improvement in physical activity, specifically for male university students. Taking into account the association between physical inactivity and mental illness, in spite of no difference in both life stress and psychological distress between two learning periods, it is recommended to carefully monitor the over-time change in mental health.

## Figures and Tables

**Table 1:** Demographic information of the participants.

	Mean ± SD or *n* (%)	Range
Age (years) ^a^	21.82 ± 3.14	18.89–39.16
Sex (female)	124 (68.51%)	
School		
Medicine	27 (14.92%)	
Chinese medicine	27 (14.92%)	
Pharmacy	24 (13.26%)	
Public health	31 (17.12%)	
Health promotion	60 (33.15%)	
Others	12 (6.63%)	
Year of studying		
1st year	47 (25.97%)	
2nd year	58 (32.04%)	
3rd year	54 (29.83%)	
4th year and higher	14 (7.73%)	
Graduate	8 (4.42%)	
Medical diagnosis (yes)	20 (11.05%)	

^a^ Data were missed in five participants.

**Table 2:** Physical and mental health during the in-class and online learning periods.

	In-Class Learning	Online Learning
Physical activity (min/week) ^a^		
Light	138.33 ± 142.18	46.42 ±56.02
Moderate	81.27 ± 98.58	35.10 ± 52.64
Vigorous	133.86 ± 178.62	65.20 ± 102.32
Total	353.46 ± 295.97	146.72 ± 143.73
Compliance ^b^	86 (47.51%)	44 (24.31%)
Psychological distress		
Total score	11.21 ± 4.63	10.81 ± 4.89
Serious mental illness	56 (30.94%)	49 (27.07%)
Student life stress		
Average subdomain score		
Physical stress	1.48 ± 0.39	1.47 ± 0.40
Interpersonal relationship	1.39 ± 0.40	1.39 ± 0.42
Academic stress	1.97 ± 0.55	1.87 ± 0.58
Environmental stress	1.86 ± 0.53	1.72 ± 0.57
Total score	67.08 ± 15.17	64.47 ± 16.22

^a^ Nineteen outliers were excluded from data analysis; ^b^ indicates the number of participants who met the WHO recommendations for physical activity.

**Table 3:** Within- and between-subject effects on physical activity.

	LPA		MVP		VPA		TPA	
	*F*_(1, 157)_	$\eta_{p}^{2}$	*F*_(1, 157)_	$\eta_{p}^{2}$	*F*_(1, 157)_	$\eta_{p}^{2}$	*F*_(1, 157)_	$\eta_{p}^{2}$
Period	17.73 ***	0.10	10.47 **	0.06	5.40 *	0.03	19.81 ***	0.11
Sex	0.91	0.01	0.38	<0.01	0.13	<0.01	0.23	<0.01
Period by Sex	1.32	0.01	1.54	0.01	4.83 *	0.03	1.86	0.01

* *p* < 0.05, ** *p* < 0.01, *** *p* < 0.001. LPA, light physical activity; MPA, moderate physical activity; MVPA, moderate-to-vigorous physical activity; PA, physical activity; TPA, total physical activity; VPA, vigorous physical activity.

**Table 4:** Within- and between-subject effects on student life distress.

	Physical Stress		Interpersonal Relationship		Academic Stress		Environmental Stress		Student Life Stress	
	*F*_(1, 176)_	$\eta_{p}^{2}$	*F*_(1, 176)_	$\eta_{p}^{2}$	*F*_(1, 176)_	$\eta_{p}^{2}$	*F*_(1, 176)_	$\eta_{p}^{2}$	*F*_(1, 176)_	$\eta_{p}^{2}$
Period	65	<0.01	0.01	<0.01	0.44	<0.01	3.00	0.02	1.29	0.01
Sex	0.88	0.01	0.02	<0.01	0.03	<0.01	0.02	<0.01	0.13	<0.01
Period by Sex	0.23	<0.01	0.09	<0.01	1.08	0.01	0.10	<0.01	0.53	<0.01

## Data Availability

The data are available on request from the corresponding author.
